# Obesity, Sarcopenia, and Outcomes in Non-Small Cell Lung Cancer Patients Treated With Immune Checkpoint Inhibitors and Tyrosine Kinase Inhibitors

**DOI:** 10.3389/fonc.2020.576314

**Published:** 2020-10-20

**Authors:** Karam Khaddour, Sandra L. Gomez-Perez, Nikita Jain, Jyoti D. Patel, Yanis Boumber

**Affiliations:** ^1^ Department of Medicine, Rosalind Franklin University of Medicine and Science, McHenry, IL, United States; ^2^ Department of Medicine, Division of Hematology and Oncology, University of Illinois at Chicago, Chicago, IL, United States; ^3^ Department of Clinical Nutrition, Rush University Medical Center, Chicago, IL, United States; ^4^ Division of Hematology/Oncology, Feinberg School of Medicine, Northwestern University, Robert H. Lurie Comprehensive Cancer Center, Chicago, IL, United States; ^5^ Institute of Fundamental Medicine and Biology, Kazan Federal University, Kazan, Russia

**Keywords:** non-small cell lung cancer, body composition, obesity, sarcopenia, tyrosine kinase inhibitor, immune checkpoint inhibitor, overall survival

## Abstract

Body composition refers to the proportional content of body fat mass and lean body mass that can lead to a continuum of different phenotypes ranging from cachectic/sarcopenic state to obesity. The heterogenetic phenotypes of body composition can contribute to formation of some cancer types and can sometimes lead to disparate outcomes. Both of these extremes of the spectrum exist in patients with non-small cell lung carcinoma (NSCLC). The discovery of new pathways that drive tumorigenesis contributing to cancer progression and resistance have expanded our understanding of cancer biology leading to development of new targeted therapies including tyrosine kinase inhibitors (TKI) and immune checkpoint inhibitors (ICI) that have changed the landscape of NSCLC treatment. However, in the new era of precision medicine, the impact of body composition phenotypes on treatment outcomes and survival is now being elucidated. In this review, we will discuss the emerging evidence of a link between body composition and outcomes in patients with NSCLC treated with TKI and ICI. We will also discuss suggested mechanisms by which body composition can impact tumor behavior and anti-tumor immunological response.

## Introduction

Body composition refers to the proportional distribution of different body mass contents amongst various compartments including adipose tissue and lean body mass. The most clinically distinct body phenotypes are obesity and sarcopenia. Obesity plays a significant role in tumorigenesis. It is believed that some cancers develop in obese individuals because of the chronic meta-inflammation associated with obesity in which an abundance of hormones and cytokines can potentiate epithelial cell proliferation and cancer formation (1). The link between obesity and cancer development in multiple tumor types has long been recognized in multiple epidemiological studies including a landmark report by the International Agency for Research on Cancer (IARC) showing an increased incidence of several solid and hematological malignancies in obese patients compared to the general population (2). Interestingly, this link is weak in the case of lung cancer (2).

In addition to its role in cancer development, obesity has now emerged as a prognostic factor that may predict cancer mortality (3–5). Obesity can be associated with either inferior or superior outcomes, depending on the cancer type. For example, systematic reviews and meta-analyses of population-based follow-up studies of obese patients with non-metastatic prostate and breast cancer across all clinical stages found a higher mortality in obese compared to normal weight patients regardless of treatment modality (4, 5). Surprisingly, obese and overweight patients with lung cancer seem to have better survival rates compared to normal weight subjects with a relative risk (RR) of 0.78 (95% CI: 0.75–0.82) in overweight and 0.79 (95% CI: 0.73–0.86) in obese patients respectively, however, this only applies to smokers (3). Moreover, a more recent Chinese study confirmed a strong inverse relationship between body mass index (BMI) and lung cancer mortality, which was again seen primarily in smokers (6).

The favorable effect of obesity on mortality in patients with non-small cell lung cancer (NSCLC) which is the most common type of lung cancer has been demonstrated in patients with local and metastatic disease who received different treatment modalities including surgery, radiation, and chemotherapy (7, 8). For example, Yang et al. demonstrated that obesity was associated with longer survival in lung cancer patients (N= 14,751) which was consistent among all stages (local, regional and distant) (7) Furthermore, a large retrospective study showed a trend for better survival in obese patients with NSCLC treated with combination doublet chemotherapies (N= 2,585) (9).

The other clinically distinct phenotype of body composition is sarcopenia which is defined by severe reduction of lean body mass and wasting of skeletal muscle. Sarcopenia has been identified as an independent prognostic factor for mortality in some cancer types including NSCLC (10, 11). In addition, a distinct overlap syndrome of increased adipose tissue (obesity) and loss of lean body mass (sarcopenia) has been recognized as an important factor contributing to worse prognosis in some cancers including NSCLC (12). These effects of obesity on mortality and worse prognosis in the presence of sarcopenia in cancer patients have generated an unprecedented interest in the field of oncology to study the interconnection between body composition phenotypes and cancer behavior including NSCLC (which will be the focus of this review) in an attempt to solve this conundrum. What adds to the importance of analyzing this association is that lung cancer has the highest prevalence, annual incidence and mortality rates worldwide compared to all cancer types in men and women (13). Likewise, body composition phenotypes such as obesity and sarcopenia are prevalent in lung cancer patients. As an example, up to 43% of patients with NSCLC cancer can develop sarcopenia and cachectic syndrome (14) and approximately about half of NSCLC patients are considered to have a BMI >25 mg/m^2^ (considered overweight or obese) (15).

Recently, there has been a revolution in our understanding of lung cancer biology with the detection of driver mutations such as epidermal growth factor receptor (EGFR) mutation as well as the exploration of the role of immune system dysfunction in cancer progression (16–18). These discoveries have transformed the outcomes of advanced lung cancer with the development of tyrosine kinase inhibitors (TKI) targeting driver mutations such as EGFR directed TKIs and immune checkpoint inhibitors (ICI) which unleash the host immune system against tumor cells (19–24). This recent development of therapeutics in NSCLC was accompanied by a huge effort to identify patients who benefit the most from these medications given that a significant proportion of patients do not respond (25). Given the established link between different body composition and outcomes in NSCLC that was outlined by prior research, it was plausible for researchers to analyze whether different body phenotypes could be a predictive factor for response and outcomes with novel therapies.

With an evolving landscape of treatment options in advanced and metastatic lung cancer, it is imperative to further understand if body composition phenotypes can predict response and outcomes to these new classes of medications and whether there is a mechanistic effect of proportionate body components and tumor microenvironment. In this review, we first introduce the common approaches used in clinical research to estimate body composition including obesity and sarcopenia. Next, we discuss the available evidence of a biological crosstalk between different body composition phenotypes and tumor microenvironment in NSCLC. Lastly, we highlight the available studies conducted to analyze the implications of body composition phenotypes on survival outcomes in patients with NSCLC who were treated with either EGFR TKI or ICI; we also provide our recommendations on the conceptual utility of incorporating body composition calculations into prospective trials.

## Discussion

### Methods Used To Estimate Body Composition in Patients With Non-Small Cell Lung Cancer

Many measures have been used in recent and ongoing research investigating the effect of body composition phenotypes on cancer outcomes including NSCLC. The most conventional and easiest method of estimating body composition to identify obesity is through calculation of BMI which is defined by weight divided by the square of body height [kg/m^2^]. Based on the world health organization (WHO) classification, individuals can be divided into six groups to estimate the degree of obesity (**Table 1**) (26, 27). However, the complexity and heterogeneity of body composition and nutritional status might not be reflected accurately with the use of BMI alone due to its low sensitivity as indicted by discrepancies between BMI and central obesity (15, 28). In addition, calculation of BMI does not offer an accurate depiction of lean body mass which, when reduced, is considered an independent prognostic factor for high mortality in patients with NSCLC (10, 11). It has also been recognized that a subset of obese patients (defined by BMI > 30 kg/m^2^) are considered to be metabolically healthy whereby they are considered to have a favorable distribution of fat mass with a normal inflammatory profile which potentially reduces the risk incurred by diseases related to obesity such as cancer and cardiovascular disease (29). Moreover, the calculation of BMI cannot distinguish between different patterns of body fat distribution (subcutaneous versus visceral) which could lead to multiple heterogeneous obesity phenotypes that could be associated with different biology and are not accurately reflected by BMI (30). Lastly, definition of obesity based on WHO classification can vary depending on ethnicity (26). This has led to the utilization of other indicators and calculations of adipose tissue content to study their relationship with outcomes in NSCLC patients such as visceral fat mass, subcutaneous fat mass, visceral to subcutaneous ratio, and fat mass index among others (31).

**Table 1:** World health organization classification of obesity based on body mass index.

Body Mass Index (BMI) (kg/m^2^)*	Definition
<18.5	Underweight
18.5 – 24.9	Normal weight
25.0 – 29.9	Overweight (Pre-obesity)
30.0 – 34.9	Obesity class 1
35.0 – 39.9	Obesity class 2
≥40	Obesity class 3

*BMI cut off points used to define obesity can vary depending on ethnicity (26).

**Figure 1:**
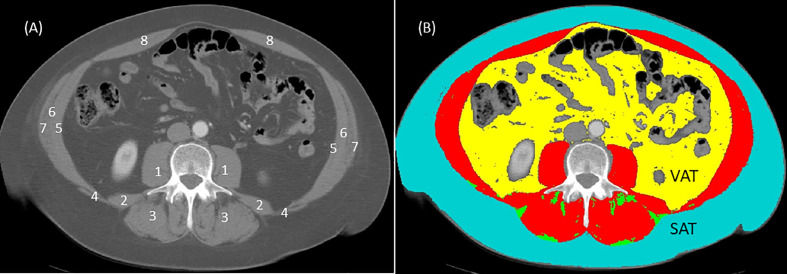
Computed tomography (CT) image at third lumbar (L3) before **(A)** and after **(B)** analysis using sliceOmatic software (Tomovision, Montreal, Quebec, Canada). In the image without coloring **(A)**, individual muscle groups are represented by numbers and these are: 1 = psoas; 2 = quadratus lumborum; 3 = erector spinae; 4 = latissimus dorsi; 5 = transversus abdominis; 6 = internal obliques; 7 = external obliques; 8 = rectus abdominis. In the image with coloring **(B)**, analysis is based on Hounsfield unit thresholds for each tissue: skeletal muscles (red) -29 to 150 HU; subcutaneous adipose tissue (SAT) in teal and intermuscular adipose tissue (IMAT) in green -30 to -190 HU; visceral adipose tissue (VAT) in yellow -50 to -150 HU; air in black -1,000 HU; bone (L3 vertebra) 400 to 4,000 HU). Skeletal muscle index (SMI) is calculated from the total surface area (cm^2^) of skeletal muscles (in red, image **(B)** normalized (divided) for height (m^2^). For example, the total L3 skeletal muscle (in red, image **(B)** for this image is 166 cm^2^ assuming that this person is female with a height of 164 cm or 2.68 m^2^ the SMI for this person = 166 cm^2^/2.68 m^2^ or 61.4 cm^2^/m^2^. Using the Prado et al. (32) cut off of SMI < 38 cm^2^/m^2^ for women, this individual would not have sarcopenia. Some researchers use single muscle groups such as the psoas muscles (1 in image **(A)** normalized for height to determine sarcopenia. Although using single muscle groups to determine sarcopenia is debatable, only the surface area for both psoas muscles (1 in image **(A)** would be used to calculate psoas muscle index (PMI). Thus, PMI = psoas muscle surface areas (cm^2^) divided by height (m^2^).

The measurement of sarcopenia is based on calculation of skeletal muscle index (SMI) (32, 33). Calculation of SMI is defined as total cross-sectional skeletal muscle mass (cm^2^) normalized by height (m^2^). Skeletal muscle mass also referred to as skeletal muscle area is derived from the total skeletal muscle mass of the eight abdominal muscles (psoas, erector spinae, quadratus lumborum, transversus abdominis, latissimus dorsi, external, and internal obliques, and rectus abdominis) measured by surface area (cm^2^) at the third lumbar (L3) landmark using a single cross-sectional computed tomography (CT) image (32, 34). The L3 landmark is visible in CT scan protocols routinely performed for diagnostic and monitoring reasons in most cancer populations: abdomen (T10-L4), chest- abdomen (T1-L4), or chest-abdomen-pelvis (T1-L5). The use of a single CT image for regional body composition analysis at L3 has been described and validated in great detail in several seminal papers (32, 34, 35). The most valuable feature of this L3 landmark is that it is linearly related to whole-body fat free mass, appendicular skeletal muscle mass and whole-body fat mass as measured by dual-energy x-ray absorptiometry (DXA) in non-cancer and cancer populations (34). In brief, the CT scanner differentiates between adipose, skeletal muscle, and other compartments like bone based on specific attenuation thresholds according to the CT unit of measurement, the Hounsfield unit (HU) scale (e.g., skeletal muscle attenuation threshold is -29 to 150 HU). Cross-sectional tissue surface areas (cm^2^) are semi-automatically determined by a medical imaging software such as SliceOmatic v 5.0 (Tomovision Montreal, Quebec, Canada) based on HU tissue-specific thresholds (34, 35) (**Figure 1**). Then, tissue boundaries are manually corrected as needed by a trained investigator following the semi-automatic analysis. Cutoff points for sarcopenia using the L3 SMI according to ethnicity, gender and BMI (sarcopenic obesity) have been validated with adverse cancer-related outcomes in several studies (32, 33).

Sarcopenic obesity is defined as low skeletal muscle mass (i.e., sarcopenia) according to a SMI cut-off such as <38.5 cm^2^/m^2^ for women and <52.4 cm^2^/m^2^ for men in the context of a BMI > 30 kg/m^2^ as published by Prado et al. (32). Similarly, sarcopenia and sarcopenic obesity can also be determined using BMI and SMI specific cut-offs for men (SMI < 43 cm^2^/m^2^ for underweight and normal weight men, SMI < 53 cm^2^/m^2^ for overweight and obese men) and for women (<41 cm^2^/m^2^ across all BMI categories) as published by Martin et al. (33). Although the Martin et al. and Prado et al. SMI cut-off values have been used extensively by other investigators, there is currently no consensus on CT-derived SMI reference cut-off values to identify sarcopenia or sarcopenic obesity in healthy and clinical populations and is an active area of research. In addition, the L3 landmark may not be ideal for assessing SMI in patients with lung cancer, including NSCLC, who often only undergo chest CT scans (i.e., L3 vertebral landmark often not visible, T1-L1). Several research studies have examined alternative chest CT scan landmarks including lumber one (L1) and two (L2) and have provided potential SMI cut-offs for sarcopenia in healthy and in lung cancer populations, however these landmarks have not been adequately tested or validated particularly in large racially diverse cancer populations (35–38). Thus, identifying an appropriate and valid single vertebral landmark from a chest CT scan and derivation of specific cut-offs at these newer landmarks to identify sarcopenia also remains an emerging area of research.

Various researchers have used single muscle groups to determine sarcopenia such as the psoas muscle index (PMI) also usually at the L3 region (39) (see **Figure 1**). The major advantage of using a single muscle group is the speed by which this analysis can be conducted in comparison to having to capture all the muscle groups at this landmark. A major criticism for the use of single muscle groups is that it does not correlate with total lumbar skeletal muscle area and thus not representative of the entirety of the lumbar muscle groups and more importantly appears to be a poor indicator of clinical outcomes in cancer populations (40, 41). Given these limitations, the use of PMI is less favorable than the well-established and validated technique using total lumbar skeletal muscle area for the calculation of SMI as previously described.

The importance of identifying sarcopenia comes from the fact that it has high prevalence across all BMI subtypes as well as having a detrimental prognostic effect on patients with NSCLC (42, 43).

As previously mentioned, an overlap syndrome of sarcopenia and obesity (sarcopenic obesity) has been identified and associated with adverse clinical implications in different cancer types, including NSCLC. For example, an observational study of patients with NSCLC who were treated with chemotherapy found that patients with sarcopenic obesity had shorter overall survival (OS) compared to obese patients without sarcopenia (44). The variable methodologies used to estimate body composition from obesity to sarcopenia and their effect on cancer progression and outcomes pose a challenge on how to compare and derive conclusions from studies in NSCLC. Nevertheless, the effort to analyze the effect of different elements of body composition and outcomes in NSCLC is of much importance, as each phenotype could have distinct biological implications on the host and the tumor.

### What Is the Effect of Obesity on Non-Small Cell Lung Cancer and Anti-Tumor Immune Response?

Obesity is considered a protective factor in both early stage and advanced NSCLC patients who are treated with surgery or chemotherapy (7, 45). This effect can be explained partially by the fact that obese patients who receive chemotherapy tend to develop less medication related toxicities leading to lower discontinuation rates of cancer treatment (46). However, the biological landscape associated with obesity seems to be more complex and is believed to play a role in cancer behavior and progression. As an example, some hormonal factors in obese patients such as leptin plasma levels can affect prognosis in NSCLC, as low leptin levels correlate with shortened OS (47). Inversely, adiponectin, which is another hormone secreted from the adipose tissue has been suggested as a factor contributing to tumor progression in NSCLC, but biological mechanisms explaining the action of this hormone are not well understood (48). Different body composition phenotypes in obese patients and their variable hormonal and inflammatory profiles have led to distinguishing obesity as “metabolically unhealthy obesity” versus “metabolically healthy obesity” whereby patients can have a high BMI consistent with obesity definition but have a favorable fat distribution with decreased systemic inflammation leading to low disease morbidity (29).

**Figure 2:**
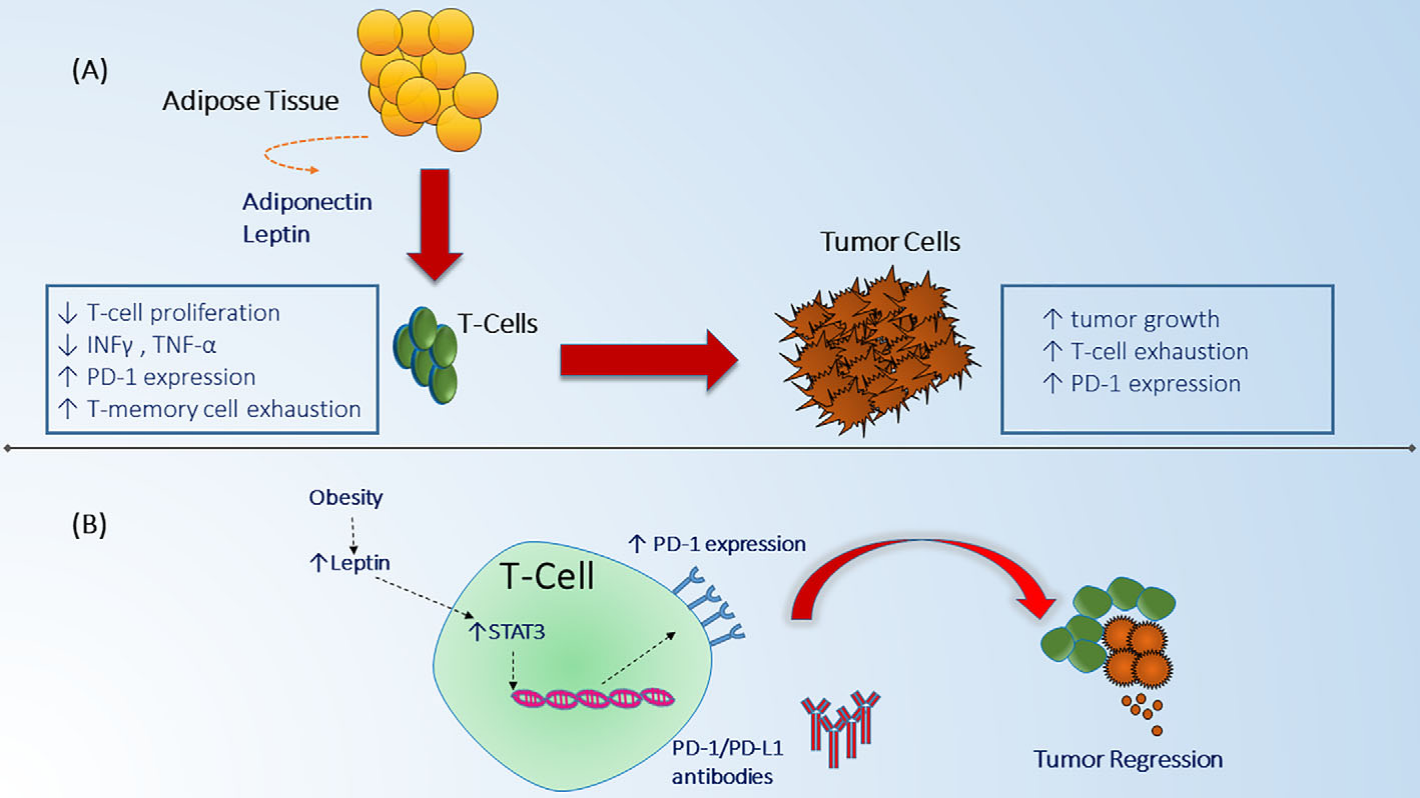
Obesity effect on immune system function and anti-tumor mechanisms. **(A)** Adipose cells in the fat tissue secrete different adipokines including adiponectine and leptin that can alter immune system function by suppressing T-cell proliferation, decreasing INF-y, TNF-a, and increasing T-cell memory dysfunction which in turn can lead to enhanced tumor escape from immune surveillance leading to tumor growth and progression (48). Obesity related tumors can as well be associated with increased expression of checkpoint proteins which have a negative regulatory effect on immune cell proliferation (50). **(B)** The mechanism by which obesity can interact with immune checkpoint receptors in the tumor microenvironment is believed to be through increased secretion of leptin which in turn increases PD-1 expression on CD8+ T-cells through STAT3 signaling (50). Increased expression of PD-1 receptors can lead to enhanced response to immune checkpoint monoclonal antibodies and immune cells mediated tumor regression (50).

The biological and immunological aspects of the inverse relationship between obesity and prognosis in NSCLC, termed “obesity paradox” can be explained through several mechanisms (49). Obesity can lead to exhaustion of T-cells resulting in increased tumor growth and it can upregulate programmed death-1 (PD-1) expression on CD8+ T-cells in tumor mice models (50). PD-1 receptors are checkpoint protein receptors present on immune cells that when bound to their respective ligand receptor can decrease anti-tumor efficacy of the host immune system against tumor cells (51). Although, the mechanism by which obesity can increase PD-1 expression has not been fully elucidated, increased levels of leptin secreted from adipose tissues has been suggested to boost cascade signaling indirectly through signal transduction and activator of transcription 3 (STAT3) which leads to upregulation of PD-1 receptors on T-cells (**Figure 2**). This can, in part, explain the improved response rates and survival noted in patients with obesity across different tumor types who are treated with ICI including PD-1/PD-L1 (programmed death ligand-1) inhibitors that target the interaction of these checkpoint receptors (52–54). Moreover, obesity can modulate other T-cell subsets such as T-regulatory (Treg) cells which function as immunosuppressant cells and when down regulated in obese patients, they can reduce production of interleukin-10 (IL-10) which leads to exacerbation of the chronic inflammatory state (55). Similarly, Treg cells have been found in the tumor microenvironment of NSCLC and lead to an inhibitory effect on effector T-cell proliferation (56). Therefore, the effect of obesity on Treg cells can play a role in modulating Treg response in NSCLC although this has not been studied yet. Another type of immune cell that is important in obesity and NSCLC are the Natural Killer (NK) cells which are responsible for innate immunity and anti-cancer function. NK cells have been shown to be impaired in patients with obesity (57). Likewise, NSCLC patients can lack the cytotoxic effect of NK cells and have defective granulation leading to decreased innate anti-tumor response (57–59). Finally, obesity can affect the balance between macrophage subtypes (M1- M2) favoring the M1 subtype (pro-inflammatory cells) over M2 (immunosuppressive and pro-tumorigenic) (60). An increase in M2 macrophages can lead to increased host immunosuppression and more aggressive tumor behavior which could theoretically explain the improved outcome profile noted in patients with NSCLC who are obese (61) (**Table 2**). This collective evidence suggests an alteration of anti-tumor immune function and the favorable outcomes in obese patients with NSCLC which can partially explain the improved outcomes noted recently with the use of ICI since this class of medications primarily affects the T-cell but also has been found to have partial mechanisms of action through other immune cells such as NK cells and macrophages (65, 66).

**Table 2:** Immune cell modulation in obesity, sarcopenia and non-small cell lung cancer.

Immune Cell Type	Modulation of immune cells, cytokines in Obesity	Modulation of immune cells, cytokines in Sarcopenia	Modulation of immune cells, cytokines in NSCLC	Reference
**T-Cell**				
**CD8+**	↑ CD8+/CD4+ ratio	↓IL-15	↑ PD-1 expression on CD8+	50, 62
	↑ expression of PD-1 on CD8+	↓CD8+		
**Treg**	Dysregulated Treg ↓IL-10	NA	↑ Treg (immunosuppression)	55, 56
	↑ Immunosuppression ↑ Inflammation		↑ CTLA-4	
**NK Cells**	↓ Cytotoxic NK cells	↓ IL-15	↓NK Degranulation	55–59, 63
		↓ NK cell activity	↓ Lytic activity	64
**Macrophage**	↓ M2 Macrophages (pro-tumorigenic)	NA	Some NSCLC ↑ M2 Macrophages which leads to immune suppression	60, 61

CTLA-4, cytotoxic lymphocyte associated antigen-4; IL-10, interleukin-10; IL-15, interleukin-15; NA, not available; NK, natural killer cells; NSCLC, non-small cell lung cancer; PD-1, programmed death-1; Treg, T- regulatory cells.

### What is the Effect of Sarcopenia on Non-Small Cell Lung Cancer and Anti-Tumor Immune Response?

**Figure 3:**
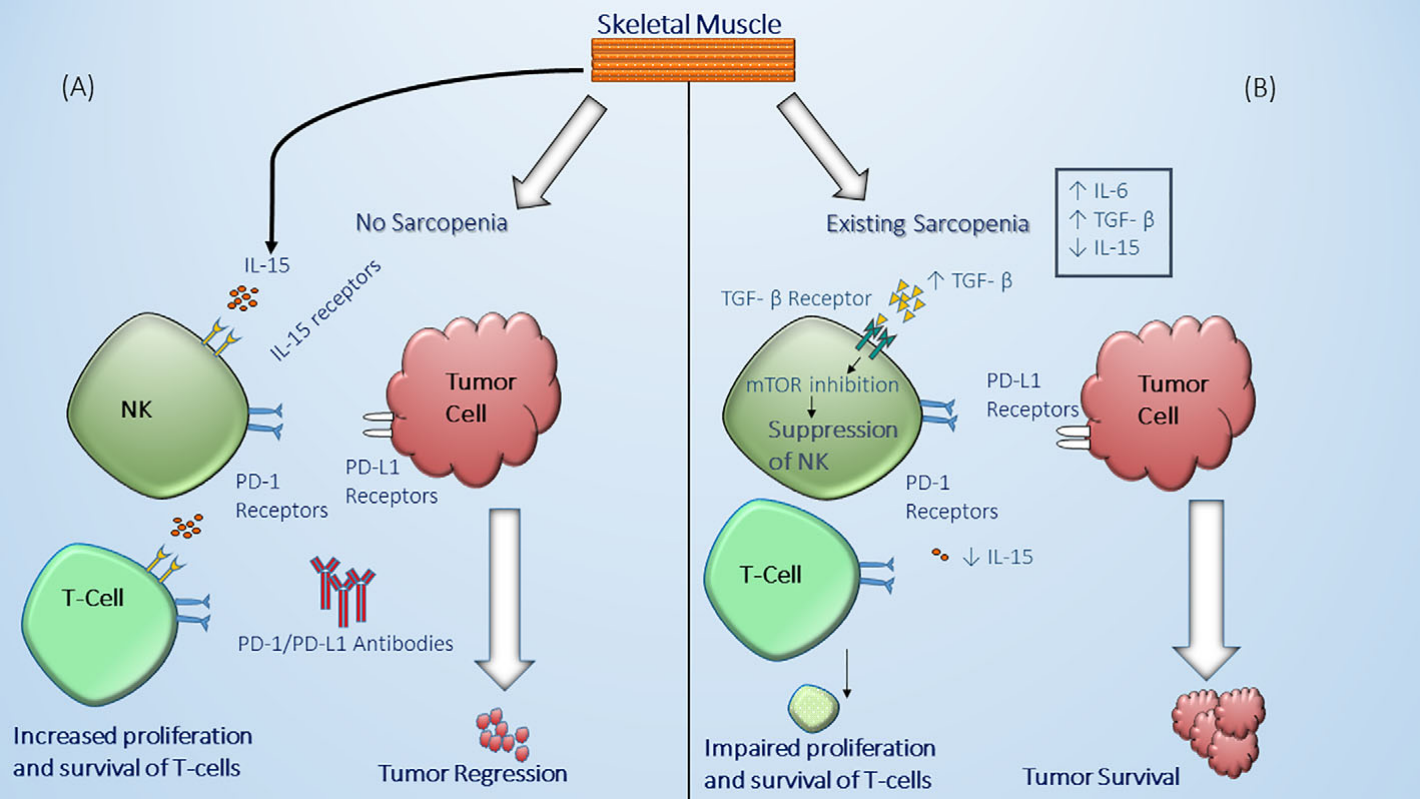
Effect of sarcopenia on immune system function and anti-tumor mechanisms. **(A)** in individuals without skeletal muscle wasting (no sarcopenia), there is sufficient production and secretion of IL-15 by skeletal muscle cells which in turn can bind to IL-15 receptors on the natural killer (NK) cell surface and T-cells leading to enhanced functional natural killer cell and proliferation and maintenance of T-cells including CD8+ T-cells against tumor (69). **(B)** In the presence of significant muscle wasting (sarcopenia), there is decreased production and secretion of IL-15 by skeletal muscle cells (69), as well as an increased chronic inflammatory status in the body associated with high levels of IL-6 and TGF-β (72–74). The latter can lead to NK suppression through mTOR inhibition leading to dysfunctional NK cell which cannot effectively eliminate malignant cells (63, 64, 75). Decreased IL-15 production can lead as well to impaired maintenance, proliferation, and survival of T-cells which are considered potential targets for immune checkpoint inhibitors.

Sarcopenia is usually considered to be a consequence of the changes accompanying malignancy such as malnutrition and alterations in the hormonal milieu including the surge of cytokines due to the presence of the tumor (67). However, the presence of sarcopenia which is manifested by reduced lean body mass including decreased skeletal muscle mass is believed to influence host immune system leading to immune senescence (68). Hence, sarcopenia can have a deleterious effect on the anti-tumor response mediated by the immune system. Also, altered cytokines levels such as elevated IL-6 and decreased IL-15 can affect immune cell function. For example, skeletal muscle cells are essential producers of IL-15 which have a positive effect on NK cell expansion, proliferation and cytotoxic effects (63, 64, 69). As such, subclasses of NK cells express PD-1 receptors and their downregulation in sarcopenia can partially explain the decreased response and worse outcomes in NSCLC patients treated with ICI (70, 71) (**Figure 3**). It should be noted that although the role of NK cells in prognosis of NSCLC patients has been suggested, the mechanism behind their role in the tumor microenvironment in lung cancer is still not well-defined (75). Similarly, it has been found that in IL-15 deficient mice models there is a reduction in the presence of CD8+ which suggests that lack of IL-15 can lead to less targetable cytotoxic T-cells by ICI (62, 76). In addition, the levels of IL-6 are increased in sarcopenic patients which can contribute to tumor growth and alters the function of immune cells including T-cell subsets (77). The possible role the pleiotropic effect of IL-6 on the immune system has tempted researchers to study the effect of targeting this proinflammatory pathway in combination with PD-1 blockade and preclinical results have shown a synergistic effect on T-cell trafficking and antitumor immunity (78). Likewise, the levels of transforming growth factor-β (TGF-β) are altered in association with sarcopenia and have been found to have a negative effect on the regulation of immune system leading to T-cell exhaustion and dysfunctional NK cells (72–74) (**Table 2**). This cumulative interconnection between sarcopenia and immune dysregulation has also been examined as in obesity to determine whether lower lean body mass impacts the response and prognosis in patients with NSCLC.

The evidence of an existing molecular and pathological relationships between different components of body composition and improved outcomes in obese and non-sarcopenic patients has been described in several retrospective studies and meta-analyses (39, 79, 80). In the following sections, we will summarize the current evidence of the impact of body composition phenotypes and cancer-related outcomes in patients with NSCLC treated with TKI-EGFR or ICI.

### Tyrosine Kinase Inhibitors, Body Composition, and Outcomes in Non-Small Cell Lung Cancer

Targeted therapy has changed the landscape of the management and prognosis in NSCLC. Targetable alterations in NSCLC include mutations in the epidermal growth factor receptor (EGFR) (accounting for up to 15% NSCLC in Europe and United States, and up to 45% in Southeast Asia) (81, 82), translocation in the anaplastic lymphoma kinase gene (ALK) (accounting for approximately 5% of cases), with a lower frequency of other mutations (ROS1, BRAF, NTRAK, and HER2) (83). Most of the phase 3 randomized controlled trials testing tyrosine kinase inhibitors (TKI) that led to approval of these targeted therapies in patients with NSCLC did not conduct subgroup analyses according to body composition (19, 20, 84). However, evidence suggests that body composition can affect outcomes in patients with NSCLC who harbor EGFR mutation and are treated with EGFR TKIs (85). A retrospective study of 630 patients with metastatic EGFR-mutant NSCLC who received either gefitinib or erlotinib (as first or later line therapy) found a relationship between higher BMI and improved progression free survival (PFS) in patients with BMI ≥ 25 kg/m^2^ compared to BMI < 18.5 kg/m^2^ (15.6 months versus 8.5 months, respectively) and OS (28.8 months versus 26.7 months, respectively) (85). Multivariate analysis in this study showed BMI as an independent risk factor in terms of PFS and OS (85). Another study used body weight (kg) to estimate PFS and OS in patients with stage IV NSCLC who were treated with gefitinib (cut off point 53 kg) (N = 138) and found a trend towards improved PFS and OS in patients with higher body weight (> 53 kg), however, it was statistically non-significant (86). Lin et al. examined the impact of weight loss prior to starting gefitinib (defined as loss of more than 5% of body weight in a 3 months period before diagnosis) on objective response rate (ORR), PFS and OS. This retrospective analysis included 75 patients and found no difference in ORR but improved PFS in patients with weight loss <5% compared to patients >5% of weight loss (12.4 months versus 7.6 months; hazard ratio [HR] 0.356, 95% confidence interval [CI] 0.212–0.596, *p <*0.001). This study also reported an improved OS in patients with <5% weight loss compared to those with >5% weight loss (28.5 months vs. 20.7 months, respectively; HR 0.408, 95% CI 0.215–0.776, *p* = 0.006) (87). In contrast, another study in patients with NSCLC who received osimertinib (N= 47) did not show any significant relationship between BMI, PFS, and OS (88). The only study that examined the association of both measures (sarcopenia and BMI) with outcomes in patients with NSCLC and EGFR mutation was a retrospective study of 167 patients who received gefitinib, erlotinib, or afatinib as a first or later line therapy (89). This study showed a BMI <18.5 kg/m^2^ to be an independent prognostic factor for worse PFS (HR 1.70 [1.03–2.81], *p*= 0.04) and OS (HR 1.72 [1.11–2.67], *p* = 0.02). However, sarcopenia defined by measurements of psoas muscle index (PMI), intermuscular adipose tissue content (IMAC), and visceral to subcutaneous adipose tissue area ratio (VSR) failed to show any effect on different outcomes (89).

Previous discrepancies in the findings of different studies challenge the theory of the effect of body composition on outcomes when NSCLC is treated with EGFR TKI. However, most studies were retrospective and observational in nature and included small sample sizes which could have undermined the relationship between body composition and efficacy of TKIs. The lack of a proposed mechanism supporting a link between body composition and signaling pathways using targetable mutations such as in EGFR mutations poses a question on whether body composition should be examined further as a marker of treatment response in this patient population. However, given the impact of body composition as an independent prognostic factor in NSCLC that have already been established with surgery and chemotherapy and its relationship with altered outcomes in patients treated with different modalities supports the need for further investigation examining these associations. Future studies examining the link between body composition and cancer-related outcomes should consider including the following parameters: better patient selection (i.e., appropriate inclusion and exclusion criteria), larger sample sizes, inclusion of newer medications like osimertinib, as well as accounting for confounding factors such as performance status and metastatic sites (90, 91).

### Immune Checkpoint Inhibitors, Body Composition, and Outcomes in Non-Small Cell Lung Cancer

The discovery of immune checkpoint molecules has revolutionized our understanding of tumor biology and resistance mechanisms (92, 93). Immune checkpoints are receptor proteins that are expressed by various immune cells that when bound to their ligands lead to suppression of effector immune cell function (94). Some inhibitory checkpoints can be related to decreased anti-tumor effect against malignant cells which usually use checkpoint ligation to escape immune surveillance (17–19). The two first targetable checkpoint receptors in tumor microenvironment discovered were cytotoxic associated lymphocyte antigen-4 (CTLA-4) and programmed death-1 (PD-1) (92, 93). Their discovery led to the development of monoclonal antibodies directed against checkpoint receptors which entered the clinical realm in NSCLC and changed the standard of care after demonstrating improved PFS and OS as first or later line therapy (21–24, 95, 96). However, not all patients retain a good response to these medications with only some patients deriving benefits with a sustained response. This has led to an effort to discover and develop predictive biomarkers to identify appropriate patient selection. Many biomarkers have emerged as predictor markers such as programmed death- ligand 1 expression (PD-L1), tumor mutational burden, and lymphocyte infiltration in tumor bed (97–99). However, adoption of these biomarkers as a conventional method to predict response and survival can be challenging given the lack of standardized definition and methodology used to quantify some of these biomarkers. Thus, the utilization of simpler available patient characteristics such as gender or body habitus seemed plausible to understand if there is any association with response to immune checkpoint inhibitors (ICI) (98–100).

The prognostic implication of body composition and survival in cancer patients has long been established in different tumor types regardless of stage or treatment approach (33, 101, 102). This concept was later adopted by researchers to examine body composition as a predictive rather than a prognostic marker for response to ICI. Perhaps the earliest link between body composition and survival when using ICI was established in melanoma patients. The work by Daly et al. demonstrated that loss of muscle mass (sarcopenia) can be associated with worse OS in melanoma patients treated with CTLA-4 inhibitors (103). This was followed by other studies that corroborated the findings as a proof of concept and were soon tested in different malignancies including NSCLC (53, 79, 104). There are two different approaches that were used to study the association between body composition and survival. The first was considering the effect of adipose tissue and obesity on survival in patients treated with ICI; while the other approach used skeletal muscle indices as a surrogate for sarcopenia. The feasibility of using such indicators to understand the interconnection was convenient given the simple methodology used to obtain these variables and soon led to several observational and comparative retrospective studies that are summarized in **Table 3** (31, 39, 52, 105–113). The findings from retrospective studies seem to be consistent with a trend towards improved PFS and OS in patients with NSCLC treated with PD-1/PD-L1 inhibitors and have been verified with meta-analyses (79, 114). Both high BMI (overweight and obese) and normal SMI (absence of sarcopenia) were associated with improved survival. It should be noted that different cut points were used to identify overweight, obese and sarcopenic patients as these indicators have different established cut points depending on variable factors such as ethnicity and health status (cancer versus no cancer) (26, 33). The use of predictive biomarkers combined with body composition status has proved a stronger correlation than using body composition alone in predicting improved PFS and OS in NSCLC patients treated with ICI in a recent large retrospective study (52). In this important paper, stronger OS/PFS benefit was observed in overweight and obese patients with PD-L1 positive tumors (defined by PD-L1 expression of > 5% of tumor cells or tumor infiltrating immune cells) compared to normal weight patients, which implies that in NSCLC, PD-L1 expressed by either tumor cells or by immune cells is critical for OS prediction in obese patients, and obesity is secondary to PD-L1 tumor status (52). Interestingly, evidence suggests that occurrence of immune related adverse events (irAEs) of any grade in different cancer types is higher in overweight and obese patients, while irAEs themselves are associated with improved PFS and OS (114, 115). Therefore, in analyzing the effect of obesity/sarcopenia on survival in NSCLC patients treated with ICI, future studies should consider analyzing the effect of body composition in different subpopulations such as patients with PD-L1 positive tumors, high tumor mutational burden, and also occurrence of irAEs. Limitations that can hinder the robustness of the previously mentioned findings in **Table 3** include the retrospective nature of the studies and small sample sizes which are both prone to sampling error and inability to detect a significant difference in specific sub-groups (such as high PD-L1 expression, irAEs).

**Table 3:** Studies on effect of body composition on tumor response and survival in patients with stage IV non-small cell lung cancer treated with immune checkpoint inhibitors.

Publication	Sample Size	Male, %	Number of PD-L1 Positive Patients	Immune Checkpoint Inhibitor	Surrogate for Body Composition	Cut-off for Surrogate	End Point	Results*	P-Value
Kichenadasse et al. (52)	1434	890 (62)	938 **	Atezolizumab	BMI	Per WHO Class	OS	Obesity vs. normal weight. HR 0.64 [CI 95%, 0.51-0.81]	P < 0.001
							PFS	Overweight and obese vs. normal weight HR 0.88 [CI 95%, 0.78-0.99]	P = 0.03
Cortellini et al. (105)	976 total with 635 NSCLC cases	663 (67.9)	NA	Pembrolizumab, Nivolumab, Atezolizumab	BMI	Overweight/ obese >= 25 vs. non-overweight <25	ORR	41.3 % vs 20.9%	P < 0.0001
							TTF	9.3 [95% CI: 8.1-11.6] vs 3.6 [95% CI: 3.2 - 4.1] months HR= 0.51 [95% CI: 0.44 – 0.60]	P < 0.0001
							PFS	11.7 [95% CI: 9.4 – 15] vs 3.7 [95% CI: 3.2 – 4.1] months HR= 0.46 [95%CI: 0.39 – 0.54]	P < 0.0001
							OS	26.6 [95% CI: 21.4 – 36.8] vs 6.6 [95% CI: 5.8 – 8.5] months HR= 0.33 [95%CI: 0.28 – 0.41]	P < 0.0001
Ichihara et al. (106)	Cohort 1: 84	68 (80.9)	84 ^***^	Pembrolizumab	BMI	22	ORR	(evaluated in 74 pts.) 0% complete response 44.6% -partial response, 32.4%- stable disease, 23%- progressive disease	
							PFS	7.3 vs. 4.7 months (HR): 0.94; 95 % CI: 0.53–1.65	P = 0.84
							OS	NR vs. 17 months HR: 0.67; 95 % CI: 0.32–1.40	P = 0.29
	Cohort 2: 429	338 (78.7)	45	Pembrolizumab, Nivolumab, Atezolizumab			ORR	(evaluated 403 pts.) 1.5% complete response, 23.3% partial response, 36.2% stable disease, 3% progressive disease	
							PFS	3.7 vs 2.8 months HR: 0.79; 95 % CI: 0.64–0.98	P = 0.036
							OS	15.4 vs 13.5 months HR: 0.73; 95 % CI: 0.57–0.95	P = 0.021
							High PDL-1 and High BMI vs Low PDL-1 and Low BMI	PFS: 17 vs 3.5 months	P = 0.007
								OS: NR vs 16.1 months	P = 0.031
Magri et al. (107)	46	28 (60.87)	NA	Nivolumab	Weight loss	Weight loss > 5% prior to therapy vs weight loss <5%	OS	2 vs 10 months	P = 0.0076
Popinat et al. (31)	55	41 (75)	13 ^****^	Nivolumab	SCFM	5 kg/m^2^	1-year OS	HR: 0.75	P = 0.006
Minami et al. (108)	74	48 (64.8)	28 ^*****^	Nivolumab, Pembrolizumab, Atezolizumab	BMI,	BMI cutoff point 18.5 Higher BMI vs lower BMI	OS	15.8 vs. 3.3 months HR = 1.83 (0.79 - 4.21)	P < 0.01
							PFS	No significant difference	-
					IMAC	Men: 0.358 Women: 0.229	OS	Low IMAC favorable for OS (HR 0.43, 95% CI 0.18 - 0.998)	P = 0.0496
							PFS	No significant difference	-
Shiroyama et al. (39)	42	26 (61.9)	NA	Nivolumab, Pembrolizumab	PMI Sarcopenia vs non-sarcopenia	Male: 6.36 cm^2^/m^2^ Female: 3.92 cm2/m2	PFS	2.1 vs 6.8 months	P= 0.004
							Overall response rate	9.1 % vs. 40%	P = 0.025
Nishioka et al. (109)	38	26 (68.4)	16 ^****^	Nivolumab, Pembrolizumab	Psoas Muscle Major Area change Sarcopenia vs non-sarcopenia	Change of equal or more than 10%	ORR	0 % versus 41%	P = 0.0154
							PFS	47 vs. 204 days [CI 23-76] vs [CI 59-NA]	P = 0.00186
Katayama et al. (110)	35	24 (68.6)	22****	Pembrolizumab, Nivolumab, Atezolizumab	BMI	>20	PFS	HR 0.43 [CI 95%, 0.19-0.95]	P = 0.036
							OS	No significant findings	-
Tsukagoshi etr al. (111)	30	23 (76.7)	NA	Nivolumab	SMI	Male: 6.36 cm2/m2. Female 3.92 cm2/m2	PFS	7.5 vs 2.8 months	P = 0.008
							OS	25 vs. 10 months	P = 0.03
							Partial response	35.3% vs 0%	P = n/a
Roch et al. (112)	142	93 (65.5)	56 ^***^ This cut off was only for those with pembrolizumab as first line	Pembrolizumab, Nivolumab	SMI Sarcopenia vs no-sarcopenia	Male: 52.4 cm^2^/m^2^ Female: 38.5 cm2/m2	PFS	2.3 vs 4.1 months	P = 0.56
							OS	7.6 vs. 12.6 months	P = 0.08
					Evolving Sarcopenia	(SMI) loss of ≥ 5%. Similar to definition of cachexia	PFS	2.3 vs 5.1 months	P = 0.04
							OS	11.2 vs 15.2 months	P = 0.07
Takada et al. (113)	103	84 (81.6)	25***	Nivolumab, pembrolizumab	SMI Low SMI vs. high SMI	Male: 25.63 cm2/m2 Female: 21.73 cm2/m2	PFS	HR 1.6 [CI 95%, 1.02- 2.50]	P = 0.0399
							OS	HR 2.04 [CI 95%, 1.14- 3.63	P = 0.0155
					BMI (univariate analysis)	Male: 21.9 Female 19.8	PFS	Not significant HR 1.20 (0.78–1.86)	P = 0.4047
							OS	HR 1.88 (1.09–3.27)	P = 0.0243
							RR	No effect of SMI or BMI on response rate	P = 0.0117

* Results reported comparing the higher than cut point group to the lower than cut point group; results are reported as either median PFS, OS or hazard ratios with confidence intervals.

** PD-L1 positivity identified by ≥5%

*** PD-L1 positivity identified by ≥ 50%

**** PD-L1 >1%

***** Tumor proportion score > 1%

Results are reported across different tumor types of which the majority were non-small cell lung cancer.

BMI, body mass index; CI, confidence interval; HR, hazard ratio; IMAC, Intermuscular adipose content; NA, not available; NR, not reached; PD-L1, programmed death-ligand-1; PFS, progression free survival; PMI, psoas muscle index; NSCLC, non-small cell lung cancer; ORR, objective response rate; OS, overall survival; SMI, skeletal muscle index; SCFM, sub-cutaneous fat mass; TTF, time to treatment failure; WHO, world health organization.

### How to Implement Body Composition Phenotypes in Designing Future Clinical Trials for Patients With Advanced Non-Small Cell Lung Cancer?

Current effort in the management of NSCLC is focused on targeting pathways involved in immune surveillance against tumor cells as well as developing novel drugs against resistant mutations that emerge after exposure to specific targeted therapy. Alongside this effort, it appears to be important to further identify subpopulations who will derive the best benefit. Given the emerging evidence of a crosstalk between different body compositions and cancer biology it will be important to incorporate subgroup analysis in prospective clinical trials when testing current available medication or novel therapies in NSCLC. This would help determine if body composition phenotypes could serve as predictive indicators for the implemented therapies or whether they serve as prognostic factors in NSCLC.

Another area of interest for future research is the changing landscape of cytokine production in obese and sarcopenic NSCLC patient categories and their effect on cancer biology and whether supplementary targeting of specific inflammatory or cytokine pathways could augment the response to immunotherapy. For instance, administration of IL-15 which has been shown to boost anti-tumor immunity *in vitro* (116). Lastly, it would be intriguing to analyze life style modification such as modulation of nutritional status and exercise or medical interventions to stabilize components of body composition such as lean body mass or adipose tissue and their effect on body composition balance and the outcomes in NSCLC when treated with ICI and other novel therapies. The impact of exercise in improving outcomes has already been established although no focus was put on weight changes as a response to therapy (117). Another example, is the use of anamorelin a ghrelin receptor agonist which maintains lean body mass and has been tested previously for the treatment of cachexia-sarcopenia syndrome in NSCLC (118).

## Conclusion

In conclusion, the study of body composition as a predictive marker in NSCLC patients treated with novel immune and targeted therapies is an area of compelling interest. Future studies should focus on incorporating subgroup analysis in large prospective trials to better analyze this association. Given that in several studies, obesity plays predictive role among smokers or primarily in PD-L1 positive NSCLC tumors, further studies focusing on BMI among these subsets are warranted. Inclusion of newer promising biomarkers such as type of EGFR mutations, PD-L1 expression and tumor mutational burden (TMB) in combination with body composition seems plausible. Unifying the definitions and cut points of different surrogate indicators of obesity or sarcopenia can be challenging but would improve our understanding of the effect of obesity and sarcopenia on survival in non-small cell lung cancer patients in the era of precision medicine.

## Author Contributions

KK conceptualized the idea of the manuscript with supervision from YB. KK performed literature search and wrote the manuscript in consultation with YB. KK provided **Figures 2**, **3** and constructed **Tables 1**–**3**. SG-P wrote the section *Methods Used in Estimating Body Composition* and provided **Figure 1**. NJ verified the information mentioned in the manuscript and contributed to **Tables 2**, **3**. JP and YB supervised the project. All authors contributed to the article and approved the submitted version.

## Funding

SG-P is supported by NCI 5R01CA204808-03. YB is supported by NIH R21 CA223394 and NIH R01 CA218802 grants and by the Russian Government Program for Competitive Growth of Kazan Federal University; YB and JP were supported by the NCI Core Grant P30 CA060553 (to Robert H Lurie Comprehensive Cancer Center at Northwestern University).

## Conflict of Interest

The authors declare that the research was conducted in the absence of any commercial or financial relationships that could be construed as a potential conflict of interest.

## Abbreviations

ALK, anaplastic lymphoma kinase gene; BMI, body mass index; CI, confidence interval; cm, centimeter; CT, computed tomography; CTLA-4, cytotoxic associated lymphocyte antigen-4; DEXA, dual-energy x-ray absorptiometry; EGFR, epidermal growth factor receptor; HR, hazard ratio; HU, Hounsfield unit; IARC, international agency for research on cancer; ICI, immune checkpoint inhibitor; IL-6, interleukin-6; IL-10, interleukin-10; IL-15, interleukin-15; IMAC, Intermuscular adipose content; irAEs, immune related adverse events; kg, kilogram; L3, third lumbar; m, meter; NA, not available; NK, natural killer; NR, not reached; PD-1, programmed death 1; PD-L1, programmed death-ligand-1; PFS, progression free survival; PMI, psoas muscle index; N, number; NSCLC, non-small cell lung cancer; ORR, objective response rate; OS, overall survival; SMI, skeletal muscle index; SCFM, sub-cutaneous fat mass; STAT3, signal transduction and activator of transcription 3; TGF-β, transforming growth factor-β; TKI, tyrosine kinase inhibitor; Treg, T-regulatory; TTF, time to treatment failure; VSR, visceral to subcutaneous adipose tissue area ratio; WHO, world health organization.

## References

[1] Himber C Delphan M Scherer D Bowers LW Hursting S Ulrich CM Signals from the adipose microenvironment and the obesity-cancer link: a systematic review. Cancer Prev Res (Phila) (2017) 10(9):494–506. 10.1158/1940-6207.CAPR-16-0322 28864539 PMC5898450

[2] Lauby-Secretan B Scoccianti C Loomis D Grosse Y Bianchini F Straif K Body Fatness and Cancer-Viewpoint of the IARC Working Group. N Engl J Med (2016) 375(8):794–8. 10.1056/NEJMsr1606602 PMC6754861 27557308

[3] Calle EE Rodriguez C Walker-Thurmond K Thun MJ Overweight, obesity, and mortality from cancer in a prospectively studied cohort of U.S. adults. N Engl J Med (2003) 348:1625–38. 10.1056/NEJMoa021423 12711737

[4] Cao Y Ma J Body mass index, prostate cancer-specific mortality, and biochemical recurrence: a systematic review and meta-analysis. Cancer Prev Res (Phila) (2011) 4(4):486–501. 10.1158/1940-6207.CAPR-10-0229 21233290 PMC3071449

[5] Chan DS Vieira AR Aune D Bandera EV Greenwood DC McTiernan A Body mass index and survival in women with breast cancer—systematic literature review and meta-analysis of 82 follow-up studies. Ann Oncol (2014) 25:1901–14. 10.1093/annonc/mdu042 PMC4176449 24769692

[6] Yang L Yang G Zhou M Smith M Ge H Boreham J Body mass index and mortality from lung cancer in smokers and nonsmokers: a nationally representative prospective study of 220,000 men in China. Int J Cancer (2009) 125(9):2136–43. 10.1002/ijc.24527 19585493

[7] Yang R Cheung MC Pedroso FE Byrne MM Koniaris LG Zimmers TA Obesity and weight loss at presentation of lung cancer are associated with opposite effects on survival. J Surg Res (2011) 170:e75–83. 10.1016/j.jss.2011.04.061 21704331 PMC3154461

[8] Ferguson MK Im HK Watson S Johnson E Wigfield CH Vigneswaran WT Association of body mass index and outcomes after major lung resection. Eur J Cardiothorac Surg (2014) 45(4):e94–9; discussion e99. 10.1093/ejcts/ezu008 24504655 PMC4402370

[9] Dahlberg SE Schiller JH Bonomi PB Sandler AB Brahmer JR Ramalingam SS Body mass index and its association with clinical outcomes for advanced non–small-cell lung cancer patients enrolled on Eastern Cooperative Oncology Group clinical trials. J Thorac Oncol (2013) 8(9):1121–7. 10.1097/JTO.0b013e31829cf942 PMC3763835 23887169

[10] Jeong SM Lee DH Giovannucci EL Predicted lean body mass, fat mass and risk of lung cancer: prospective US cohort study. Eur J Epidemiol (2019) 34(12):1151–60. 10.1007/s10654-019-00587-2 PMC7504685 31754943

[11] Buentzel J Heinz J Bleckmann A Bauer C Röver C Bohnenberger H Sarcopenia as Prognostic Factor in Lung Cancer Patients: A Systematic Review and Meta-analysis. Anticancer Res (2019) 39(9):4603–12. 10.21873/anticanres.13640 31519557

[12] Suzuki Y Okamoto T Fujishita T Katsura M Akamine T Takamori S Clinical implications of sarcopenia in patients undergoing complete resection for early non-small cell lung cancer. Lung Cancer (2016) 101:92–7. 10.1016/j.lungcan.2016.08.007 27794415

[13] Bray F Ferlay J Soerjomataram I Siegel RL Torre LA Jemal A Global cancer statistics 2018: GLOBOCAN estimates of incidence and mortality worldwide for 36 cancers in 185 countries. CA Cancer J Clin (2018) 68(6):394–424. 10.3322/caac.21492 30207593

[14] Yang M Shen Y Tan L Li W Prognostic Value of Sarcopenia in Lung Cancer: A Systematic Review and Meta-analysis. Chest (2019) 156(1):101–11. 10.1016/j.chest.2019.04.115 31128115

[15] Yu D Zheng W Johansson M Lan Q Park Y White E Overall and Central Obesity and Risk of Lung Cancer: A Pooled Analysis. J Natl Cancer Inst (2018) 110(8):831–42. 10.1093/jnci/djx286 PMC6093439 29518203

[16] Walunas TL Lenschow DJ Bakker CY Linsley PS Freeman GJ Green JM CTLA-4 can function as a negative regulator of T cell activation. Immunity (1994) 1(5):405–13. 10.1016/1074-7613(94)90071-x 7882171

[17] Freeman GJ Long AJ Iwai Y Bourque K Chernova T Nishimura H Engagement of the PD-1 immunoinhibitory receptor by a novel B7 family member leads to negative regulation of lymphocyte activation. J Exp Med (2000) 192(7):1027–34. 10.1084/jem.192.7.1027 PMC2193311 11015443

[18] Waterhouse P Penninger JM Timms E Wakeham A Shahinian A Lee KP Lymphoproliferative disorders with early lethality in mice deficient in Ctla-4. Science (1995) 270(5238):985–8. 10.1126/science.270.5238.985 7481803

[19] Cappuzzo F Ciuleanu T Stelmakh L Cicenas S Szczésna A Juhász E Erlotinib as maintenance treatment in advanced non-small-cell lung cancer: a multicentre, randomised, placebo-controlled phase 3 study. Lancet Oncol (2010) 11:521–29. 10.1016/S1470-2045(10)70112-1 20493771

[20] Fukuoka M Wu YL Thongprasert S Sunpaweravong P Leong SS Sriuranpong V Biomarker analyses and final overall survival results from a phase III, randomized, open-label, first-line study of gefitinib versus carboplatin/paclitaxel in clinically selected patients with advanced non-small-cell lung cancer in Asia (IPASS). J Clin Oncol (2011) 29(21):2866–74. 10.1200/JCO.2010.33.4235 21670455

[21] Herbst RS Baas P Kim DW Felip E Pérez-Gracia JL Han JY Pembrolizumab versus docetaxel for previously treated, PD-L1-positive, advanced non-small-cell lung cancer (KEYNOTE-010): a randomised controlled trial. Lancet (2016) 387(10027):1540–50. 10.1016/S0140-6736(15)01281-7 26712084

[22] Rittmeyer A Barlesi F Waterkamp D Park K Ciardiello F von Pawel J Atezolizumab versus docetaxel in patients with previously treated non-small-cell lung cancer (OAK): a phase 3, open-label, multicentre randomised controlled trial. Lancet (2017) 389(10066):255–65. 10.1016/S0140-6736(16)32517-X PMC6886121 27979383

[23] Borghaei H Paz-Ares L Horn L Spigel DR Steins M Ready NE Nivolumab versus Docetaxel in Advanced Nonsquamous Non-Small-Cell Lung Cancer. N Engl J Med (2015) 373(17):1627–39. 10.1056/NEJMoa1507643 PMC5705936 26412456

[24] Brahmer J Reckamp KL Baas P Crinò L Eberhardt WE Poddubskaya E Nivolumab versus Docetaxel in Advanced Squamous-Cell Non-Small-Cell Lung Cancer. N Engl J Med (2015) 373(2):123–35. 10.1056/NEJMoa1504627 PMC4681400 26028407

[25] Haslam A Gill J Prasad V Estimation of the Percentage of US Patients With Cancer Who Are Eligible for Immune Checkpoint Inhibitor Drugs. JAMA Netw Open (2020) 3(3):e200423. 10.1001/jamanetworkopen.2020.0423 32150268 PMC7063495

[26] Razak F Anand SS Shannon H Vuksan V Davis B Jacobs R Defining obesity cut points in a multiethnic population. Circulation (2007) 115(16):2111–8. 10.1161/CIRCULATIONAHA.106.635011 17420343

[27] Organization WHO Obesity: Preventing and Managing the Global Epidemic. World Health Organization (2000). 11234459

[28] Romero-Corral A Somers VK Sierra-Johnson J Thomas RJ Collazo-Clavell ML Korinek J Accuracy of body mass index in diagnosing obesity in the adult general population. Int J Obes (Lond) (2008) 32(6):959–66. 10.1038/ijo.2008.11 PMC2877506 18283284

[29] Kovarik M Hronek M Zadak Z Clinically relevant determinants of body composition, function and nutritional status as mortality predictors in lung cancer patients. Lung Cancer (2014) 84(1):1–6. 10.1016/j.lungcan.2014.01.020 24560334

[30] Shimizu H Shimomura Y Hayashi R Ohtani K Sato N Futawatari T Serum leptin concentration is associated with total body fat mass, but not abdominal fat distribution. Int J Obes Relat Metab Disord (1997) 21(7):536–41. 10.1038/sj.ijo.0800437 9226482

[31] Popinat G Cousse S Goldfarb L Becker S Gardin I Salaün M Sub-cutaneous Fat Mass measured on multislice computed tomography of pretreatment PET/CT is a prognostic factor of stage IV non-small cell lung cancer treated by nivolumab. Oncoimmunology (2019) 8(5):e1580128. 10.1080/2162402X.2019.1580128 31069139 PMC6492978

[32] Prado CM Lieffers JR McCargar LJ Reiman T Sawyer MB Martin L Prevalence and clinical implications of sarcopenic obesity in patients with solid tumours of the respiratory and gastrointestinal tracts: a population-based study. Lancet Oncol (2008) 9(7):629–35. 10.1016/S1470-2045(08)70153-0 18539529

[33] Martin L Birdsell L MacDonald N Reiman T Clandinin MT McCargar LJ Cancer Cachexia in the Age of Obesity: Skeletal Muscle Depletion Is a Powerful Prognostic Factor, Independent of Body Mass Index. J Clin Oncol (2013) 31:1539–47. 10.1200/JCO.2012.45.2722 23530101

[34] Mourtzakis M Prado CM Lieffers JR Reiman T McCargar LJ Baracos VE A practical and precise approach to quantification of body composition in cancer patients using computed tomography images acquired during routine care. Appl Physiol Nutr Metab (2008) 33(5):997–1006. 10.1139/H08-075 18923576

[35] Shen W Punyanitya M Wang Z Gallagher D St-Onge MP Albu J Total body skeletal muscle and adipose tissue volumes: estimation from a single abdominal cross-sectional image. J Appl Physiol (1985) (2004) 97(6):2333–8. 10.1152/japplphysiol.00744.2004 15310748

[36] Derstine BA Holcombe SA Ross BE Wang NC Su GL Wang SC Skeletal muscle cutoff values for sarcopenia diagnosis using T10 to L5 measurements in a healthy US population. Sci Rep (2018) 8(1):11369. 10.1038/s41598-018-29825-5 30054580 PMC6063941

[37] Kim EY Kim YS Park I Ahn HK Cho EK Jeong YM Evaluation of sarcopenia in small-cell lung cancer patients by routine chest CT. Support Care Cancer (2016) 24(11):4721–6. 10.1007/s00520-016-3321-0 27364150

[38] Recio-Boiles A Galeas JN Goldwasser B Sanchez K Man LMW Gentzler RD Enhancing evaluation of sarcopenia in patients with non-small cell lung cancer (NSCLC) by assessing skeletal muscle index (SMI) at the first lumbar (L1) level on routine chest computed tomography (CT). Support Care Cancer (2018) 26(7):2353–9. 10.1007/s00520-018-4051-2 PMC5984123 29417293

[39] Shiroyama T Nagatomo I Koyama S Hirata H Nishida S Miyake K Impact of sarcopenia in patients with advanced non-small cell lung cancer treated with PD-1 inhibitors: A preliminary retrospective study. Sci Rep (2019) 9(1):2447. 10.1038/s41598-019-39120-6 30792455 PMC6385253

[40] Baracos VE Psoas as a sentinel muscle for sarcopenia: a flawed premise. J Cachexia Sarcopenia Muscle (2017) 8(4):527–8. 10.1002/jcsm.12221 PMC5566635 28675689

[41] Rutten IJG Ubachs J Kruitwagen RFPM Beets-Tan RGH Olde Damink SWM Van Gorp T Psoas muscle area is not representative of total skeletal muscle area in the assessment of sarcopenia in ovarian cancer. J Cachexia Sarcopenia Muscle (2017) 8(4):630–8. 10.1002/jcsm.12180 PMC5566632 28513088

[42] Baracos VE Reiman T Mourtzakis M Gioulbasanis I Antoun S Body composition in patients with non-small cell lung cancer: a contemporary view of cancer cachexia with the use of computed tomography image analysis. Am J Clin Nutr (2010) 91(4):1133S–7S. 10.3945/ajcn.2010.28608C 20164322

[43] Shachar SS Williams GR Muss HB Nishijima TF Prognostic value of sarcopenia in adults with solid tumours: A meta-analysis and systematic review. Eur J Cancer (2016) 57:58–67. 10.1016/j.ejca.2015.12.030 26882087

[44] Gonzalez MC Pastore CA Orlandi SP Heymsfield SB Obesity paradox in cancer: new insights provided by body composition. Am J Clin Nutr (2014) 99(5):999–1005. 10.3945/ajcn.113.071399 24572565

[45] Lam VK Bentzen SM Mohindra P Nichols EM Bhooshan N Vyfhuis M Obesity is associated with long-term improved survival in definitively treated locally advanced non-small cell lung cancer (NSCLC). Lung Cancer (2017) 104:52–7. 10.1016/j.lungcan.2016.11.017 28213000

[46] Kashiwabara K Yamane H Tanaka H Toxicity and prognosis in overweight and obese women with lung cancer receiving carboplatin-paclitaxel doublet chemotherapy. Cancer Invest (2013) 31(4):251–7. 10.3109/07357907.2013.784778 23607633

[47] Kerenidi T Lada M Tsaroucha A Georgoulias P Mystridou P Gourgoulianis Kl. Clinical significance of serum adipokines levels in lung cancer. Med Oncol (2013) 30:507. 10.1007/s12032-013-0507-x 23430445

[48] Boura P Loukides S Grapsa D Achimastos A Syrigos K The diverse roles of adiponectine in non-small-cell lung cancer: current data and future prespectives. Future Oncol (2015) 11:2193–203. 10.2217/fon.15.96 26235182

[49] Zhang X Liu Y Shao H Zheng X Obesity paradox in lung cancer prognosis: evolving biological insights and clinical implications. J Thoracic Oncol (2017) 12(10):1478–88. 10.1016/j.jtho.2017.07.022 28757418

[50] Wang Z Aguilar EG Luna JI Dunai C Khuat LT Le CT Paradoxical effects of obesity on T cell function during tumor progression and PD-1 checkpoint blockade. Nat Med (2019) 25(1):141–51. 10.1038/s41591-018-0221-5 PMC6324991 30420753

[51] Weber J Immune checkpoint proteins: a new therapeutic paradigm for cancer–preclinical background: CTLA-4 and PD-1 blockade. Semin Oncol (2010) 37(5):430–9. 10.1053/j.seminoncol.2010.09.005 21074057

[52] Kichenadasse G Miners JO Mangoni AA Rowland A Hopkins AM Sorich MJ Association between body mass index and overall survival with immune checkpoint inhibitor therapy for advanced non–small cell lung cancer. JAMA Oncol (2019) 6(4):512–8. 10.1001/jamaoncol.2019.5241 PMC6990855 31876896

[53] McQuade JL Daniel CR Hess KR Mak C Wang DY Rai RR Association of body-mass index and outcomes in patients with metastatic melanoma treated with targeted therapy, immunotherapy, or chemotherapy: a retrospective, multicohort analysis. Lancet Oncol (2018) 19(3):310–22. 10.1016/S1470-2045(18)30078-0 PMC5840029 29449192

[54] De Giorgi U Procopio G Giannarelli D Sabbatini R Bearz A Buti S Association of Systemic Inflammation Index and Body Mass Index with Survival in Patients with Renal Cell Cancer Treated with Nivolumab. Clin Cancer Res (2019) 25(13):3839–46. 10.1158/1078-0432.CCR-18-3661 30967420

[55] Han JM Patterson SJ Speck M Ehses JA Levings MK Insulin inhibits IL-10-mediated regulatory T cell function: implications for obesity. J Immunol (2014) 192(2):623–9. 10.4049/jimmunol.1302181 24323581

[56] Woo EY Yeh H Chu CS Schlienger K Carroll RG Riley JL Cutting edge: Regulatory T cells from lung cancer patients directly inhibit autologous T cell proliferation. J Immunol (2002) 168(9):4272–6. 10.4049/jimmunol.168.9.4272 11970966

[57] Bahr I Jahn J Zipprich A Pahlow I Spielmann J Kielstein H Impaired natural killer cell subset phenotypes in human obesity. Immunol Res (2018) 66:234–44. 10.1007/s12026-018-8989-4 PMC5899081 29560551

[58] Carrega P Morandi B Costa R Frumento G Forte G Altavilla G Natural killer cells infiltrating human nonsmall-cell lung cancer are enriched in CD56 bright CD16(-) cells and display an impaired capability to kill tumor cells. Cancer (2008) 112(4):863–75. 10.1002/cncr.23239 18203207

[59] Platonova S Cherfils-Vicini J Damotte D Crozet L Vieillard V Validire P Profound coordinated alterations of intratumoral NK cell phenotype and function in lung carcinoma. Cancer Res (2011) 71(16):5412–22. 10.1158/0008-5472.CAN-10-4179 21708957

[60] Kraakman MJ Murphy AJ Jandeleit-Dahm K Kammoun HL Macrophage polarization in obesity and type 2 diabetes: weighing down our understanding of macrophage function? Front Immunol (2014) 5:470. 10.3389/fimmu.2014.00470 25309549 PMC4176397

[61] Dai F Liu L Che G Yu N Pu Q Zhang S The number and microlocalization of tumor-associated immune cells are associated with patient’s survival time in non-small cell lung cancer. BMC Cancer (2010) 10:220. 10.1186/1471-2407-10-220 20487543 PMC2880994

[62] Kennedy MK Glaccum M Brown SN Butz EA Viney JL Embers M Reversible defects in natural killer and memory CD8 t cell lineages in interleukin 15-deficient mice. J Exp Med (2000) 191(5):771–80. 10.1084/jem.191.5.771 PMC2195858 10704459

[63] Carson WE Giri JG Lindemann MJ Linett ML Ahdieh M Paxton R Interleukin (IL) 15 is a novel cytokine that activates human natural killer cells via components of the IL-2 receptor. J Exp Med (1994) 180(4):1395–403. 10.1084/jem.180.4.1395 PMC2191697 7523571

[64] Van den Bergh J Willemen Y Lion E Van Acker H De Reu H Anguille S Transpresentation of interleukin-15 by IL-15/IL-15Rα mRNA-engineered human dendritic cells boosts antitumoral natural killer cell activity. Oncotarget (2015) 6(42):44123–33. 10.18632/oncotarget.6536 PMC4792546 26675759

[65] Dong W Wu X Ma S Wang Y Nalin AP Zhu Z The Mechanism of Anti-PD-L1 Antibody Efficacy against PD-L1-Negative Tumors Identifies NK Cells Expressing PD-L1 as a Cytolytic Effector. Cancer Discov (2019) 9(10):1422–37. 10.1158/2159-8290.CD-18-1259 PMC7253691 31340937

[66] Xiong H Mittman S Rodriguez R Moskalenko M Pacheco-Sanchez P Yang Y Anti-PD-L1 Treatment Results in Functional Remodeling of the Macrophage Compartment. Cancer Res (2019) 79(7):1493–506. 10.1158/0008-5472.CAN-18-3208 30679180

[67] Argilés JM Busquets S Felipe A López-Soriano FJ Molecular mechanisms involved in muscle wasting in cancer and ageing: cachexia versus sarcopenia. Int J Biochem Cell Biol (2005) 37(5):1084–104. 10.1016/j.biocel.2004.10.003 15743680

[68] Nelke C Dziewas R Minnerup J Meuth SG Ruck T Skeletal muscle as potential central link between sarcopenia and immune senescence. EBioMedicine (2019) 49:381–8. 10.1016/j.ebiom.2019.10.034 PMC6945275 31662290

[69] Nielsen AR Mounier R Plomgaard P Mortensen OH Penkowa M Speerschneider T Expression of interleukin-15 in human skeletal muscle effect of exercise and muscle fibre type composition. J Physiol (2007) 584(Pt 1):305–12. 10.1113/jphysiol.2007.139618 PMC2277063 17690139

[70] Moretta L Bottino C Pende D Vitale M Mingari MC Moretta A Different checkpoints in human NK-cell activation. Trends Immunol (2004) 25(12):670–6. 10.1016/j.it.2004.09.008 15530838

[71] Chiossone L Vivier E Immune checkpoints on innate lymphoid cells. J Exp Med (2017) 214(6):1561–3. 10.1084/jem.20170763 PMC5461009 28515074

[72] Burks TN Cohn RD Role of TGF-β signaling in inherited and acquired myopathies. Skelet Muscle (2011) 1(1):19. 10.1186/2044-5040-1-19 21798096 PMC3156642

[73] Mariathasan S Turley SJ Nickles D Castiglioni A Yuen K Wang Y TGFβ attenuates tumour response to PD-L1 blockade by contributing to exclusion of T cells. Nature (2018) 554(7693):544–8. 10.1038/nature25501 PMC6028240 29443960

[74] Viel S Marçais A Guimaraes FS Loftus R Rabilloud J Grau M TGF-β inhibits the activation and functions of NK cells by repressing the mTOR pathway. Sci Signal (2016) 9(415):ra19. 10.1126/scisignal.aad1884 26884601

[75] Carrega P Ferlazzo G Natural Killers Are Made Not Born: How to Exploit NK Cells in Lung Malignancies. Front Immunol (2017) 8:277. 10.3389/fimmu.2017.00277 28348567 PMC5346886

[76] Conlon KC Lugli E Welles HC Rosenberg SA Fojo AT Morris JC Redistribution, hyperproliferation, activation of natural killer cells and CD8 T cells, and cytokine production during first-in-human clinical trial of recombinant human interleukin-15 in patients with cancer. J Clin Oncol (2015) 33(1):74–82. 10.1200/JCO.2014.57.3329 25403209 PMC4268254

[77] Maggio M Guralnik JM Longo DL Ferrucci L Interleukin-6 in aging and chronic disease: a magnificent pathway. J Gerontol A Biol Sci Med Sci (2006) 61(6):575–84. 10.1093/gerona/61.6.575 PMC2645627 16799139

[78] Tsukamoto H Fujieda K Miyashita A Fukushima S Ikeda T Kubo Y Combined Blockade of IL6 and PD-1/PD-L1 Signaling Abrogates Mutual Regulation of Their Immunosuppressive Effects in the Tumor Microenvironment. Cancer Res (2018) 78(17):5011–22. 10.1158/0008-5472.CAN-18-0118 29967259

[79] Xu H Cao D He A Ge W The prognostic role of obesity is independent of sex in cancer patients treated with immune checkpoint inhibitors: A pooled analysis of 4090 cancer patients. Int Immunopharmacol (2019) 74:105745. 10.1016/j.intimp.2019.105745 31302449

[80] Cortellini A Verna L Porzio G Bozzetti F Palumbo P Masciocchi C Predictive value of skeletal muscle mass for immunotherapy with nivolumab in non-small cell lung cancer patients: A “hypothesis-generator” preliminary report. Thorac Cancer (2019) 10(2):347–51. 10.1111/1759-7714.12965 PMC6360197 30600905

[81] Kawaguchi T Koh Y Ando M Ito N Takeo S Adachi H Prospective Analysis of Oncogenic Driver Mutations and Environmental Factors: Japan Molecular Epidemiology for Lung Cancer Study. J Clin Oncol (2016) 34(19):2247–57. 10.1200/JCO.2015.64.2322 27161973

[82] Han B Tjulandin S Hagiwara K Normanno N Wulandari L Laktionov K EGFR mutation prevalence in Asia-Pacific and Russian patients with advanced NSCLC of adenocarcinoma and non-adenocarcinoma histology: The IGNITE study. Lung Cancer (2017) 113:37–44. 10.1016/j.lungcan.2017.08.021 29110846

[83] Chia PL Mitchell P Dobrovic A John T Prevalence and natural history of ALK positive non-small-cell lung cancer and the clinical impact of targeted therapy with ALK inhibitors. Clin Epidemiol (2014) 6:423–32. 10.2147/CLEP.S69718 PMC4242069 25429239

[84] Ding Z Chen Y Hong Y Fu Y Tong L Li Q Obesity has an impact on the efficacy of EGFR-TKI in nonsmall cell lung cancer patients harboring EGFR mutation: A real-world study. Ann Oncol (2019) 30(suppl_2):ii38–68. 10.1093/annonc/mdz063 30407504

[85] Park S Park S Lee SH Suh B Keam B Kim TM Nutritional status in the era of target therapy: poor nutrition is a prognostic factor in non-small cell lung cancer with activating epidermal growth factor receptor mutations. Korean J Intern Med (2016) 31(6):1140–9. 10.3904/kjim.2015.062 PMC5094922 27017943

[86] Imai H Kuwako T Kaira K Masuda T Miura Y Seki K Evaluation of gefitinib efficacy according to body mass index, body surface area, and body weight in patients with EGFR-mutated advanced non-small cell lung cancer. Cancer Chemother Pharmacol (2017) 79(3):497–505. 10.1007/s00280-016-3232-2 28168310 PMC5344961

[87] Lin L Zhao J Hu J Huang F Han J He Y Impact of Weight Loss at Presentation on Survival in Epidermal Growth Factor Receptor Tyrosine Kinase Inhibitors (EGFR-TKI) Sensitive Mutant Advanced Non-small Cell Lung Cancer (NSCLC) Treated with First-line EGFR-TKI. J Cancer (2018) 9(3):528–34. 10.7150/jca.22378 PMC5820920 29483958

[88] Ono T Igawa S Ozawa T Kasajima M Ishihara M Hiyoshi Y Evaluation of osimertinib efficacy according to body surface area and body mass index in patients with non-small cell lung cancer harboring an EGFR mutation: A prospective observational study. Thorac Cancer (2019) 10(4):880–9. 10.1111/1759-7714.13018 PMC6590234 30821083

[89] Minami S Ihara S Nishimatsu K Komuta K Low Body Mass Index Is an Independent Prognostic Factor in Patients With Non-Small Cell Lung Cancer Treated With Epidermal Growth Factor Receptor Tyrosine Kinase Inhibitor. World J Oncol (2019) 10(6):187–98. 10.14740/wjon1244 PMC6940038 31921375

[90] Kawaguchi T Takada M Kubo A Matsumura A Fukai S Tamura A Performance status and smoking status are independent favorable prognostic factors for survival in non-small cell lung cancer: a comprehensive analysis of 26,957 patients with NSCLC. J Thorac Oncol (2010) 5(5):620–30. 10.1097/JTO.0b013e3181d2dcd9 20354456

[91] Tamura T Kurishima K Nakazawa K Kagohashi K Ishikawa H Satoh H Specific organ metastases and survival in metastatic non-small-cell lung cancer. Mol Clin Oncol (2015) 3(1):217–21. 10.3892/mco.2014.410 PMC4251107 25469298

[92] Brunet JF Denizot F Luciani MF Roux-Dosseto M Suzan M Mattei MG A new member of the immunoglobulin superfamily–CTLA-4. Nature (1987) 328(6127):267–70. 10.1038/328267a0 3496540

[93] Agata Y Kawasaki A Nishimura H Ishida Y Tsubata T Yagita H Expression of the PD-1 antigen on the surface of stimulated mouse T and B lymphocytes. Int Immunol (1996) 8(5):765–72. 10.1093/intimm/8.5.765 8671665

[94] Azoury SC Straughan DM Shukla V Immune Checkpoint Inhibitors for Cancer Therapy: Clinical Efficacy and Safety. Curr Cancer Drug Targ (2015) 15(6):452–62. 10.2174/156800961506150805145120 26282545

[95] Reck M Rodríguez-Abreu D Robinson AG Hui R Csőszi T Fülöp A Pembrolizumab versus Chemotherapy for PD-L1-Positive Non-Small-Cell Lung Cancer. N Engl J Med (2016) 375(19):1823–33. 10.1056/NEJMoa1606774 27718847

[96] Gandhi L Rodríguez-Abreu D Gadgeel S Esteban E Felip E De Angelis F Pembrolizumab plus Chemotherapy in Metastatic Non-Small-Cell Lung Cancer. N Engl J Med (2018) 378(22):2078–92. 10.1056/NEJMoa1801005 29658856

[97] Arora S Velichinskii R Lesh RW Ali U Kubiak M Bansal P Existing and Emerging Biomarkers for Immune Checkpoint Immunotherapy in Solid Tumors. Adv Ther (2019) 36(10):2638–78. 10.1007/s12325-019-01051-z PMC6778545 31410780

[98] Willis C Fiander M Tran D Korytowsky B Thomas JM Calderon F Tumor mutational burden in lung cancer: a systematic literature review. Oncotarget (2019) 10(61):6604–22. 10.18632/oncotarget.27287 PMC6859921 31762941

[99] Brambilla E Le Teuff G Marguet S Lantuejoul S Dunant A Graziano S Prognostic Effect of Tumor Lymphocytic Infiltration in Resectable Non-Small-Cell Lung Cancer. J Clin Oncol (2016) 34(11):1223–30. 10.1200/JCO.2015.63.0970 PMC4872323 26834066

[100] Conforti F Pala L Bagnardi V De Pas T Martinetti M Viale G Cancer immunotherapy efficacy and patients’ sex: a systematic review and meta-analysis. Lancet Oncol (2018) 19(6):737–46. 10.1016/S1470-2045(18)30261-4 29778737

[101] Antoun S Lanoy E Iacovelli R Albiges-Sauvin L Loriot Y Merad-Taoufik M Skeletal muscle density predicts prognosis in patients with metastatic renal cell carcinoma treated with targeted therapies. Cancer (2013) 119(18):3377–84. 10.1002/cncr.28218 23801109

[102] Sabel MS Lee J Cai S Englesbe MJ Holcombe S Wang S Sarcopenia as a prognostic factor among patients with stage III melanoma. Ann Surg Oncol (2011) 18(13):3579–85. 10.1245/s10434-011-1976-9 21822551

[103] Daly LE Power DG O’Reilly á Donnellan P Cushen SJ O'Sullivan K The impact of body composition parameters on ipilimumab toxicity and survival in patients with metastatic melanoma. Br J Cancer (2017) 116(3):310–7. 10.1038/bjc.2016.431 PMC5294486 28072766

[104] Richtig G Hoeller C Wolf M Wolf I Rainer BM Schulter G Body mass index may predict the response to ipilimumab in metastatic melanoma: An observational multi-centre study. PLoS One (2018) 13(10):e0204729. 10.1371/journal.pone.0204729 30273398 PMC6166940

[105] Cortellini A Bersanelli M Buti S Cannita K Santini D Perrone F A multicenter study of body mass index in cancer patients treated with anti-PD-1/PD-L1 immune checkpoint inhibitors: when overweight becomes favorable. J Immuno Ther Cancer (2019) 7(1):57. 10.1186/s40425-019-0527-y. PMC6391761 30813970

[106] Ichihara E Harada D Inoue K Sato K Hosokawa S Kishino D The impact of body mass index on the efficacy of anti-PD-1/PD-L1 antibodies in patients with non-small cell lung cancer. Lung Cancer (2020) 139:140–5. 10.1016/j.lungcan.2019.11.011 31786476

[107] Magri V Gottfried T Di Segni M Urban D Peled M Daher S Correlation of body composition by computerized tomography and metabolic parameters with survival of nivolumab-treated lung cancer patients. Cancer Manag Res (2019) 11:8201–7. 10.2147/CMAR.S210958 PMC6733251 31564979

[108] Minami S Ihara S Tanaka T Komuta K Sarcopenia and Visceral Adiposity Did Not Affect Efficacy of Immune-Checkpoint Inhibitor Monotherapy for Pretreated Patients With Advanced Non-Small Cell Lung Cancer. World J Oncol (2020) 11(1):9–22. 10.14740/wjon1225 32095185 PMC7011908

[109] Nishioka N Uchino J Hirai S Katayama Y Yoshimura A Okura N Association of Sarcopenia with and Efficacy of Anti-PD-1/PD-L1 Therapy in Non-Small-Cell Lung Cancer. J Clin Med (2019) 8(4):450. 10.3390/jcm8040450 PMC6518257 30987236

[110] Katayama Y Shimamoto T Yamada T Takeda T Yamada T Shiotsu S Retrospective Efficacy Analysis of Immune Checkpoint Inhibitor Rechallenge in Patients with Non-Small Cell Lung Cancer. J Clin Med (2019) 9(1):102. 10.3390/jcm9010102 PMC7019787 31906082

[111] Tsukagoshi M Yokobori T Yajima T Maeno T Shimizu K Mogi A Skeletal muscle mass predicts the outcome of nivolumab treatment for non-small cell lung cancer. Med (Baltimore) (2020) 99(7):e19059. 10.1097/MD.0000000000019059 PMC7035054 32049805

[112] Roch B Coffy A Jean-Baptiste S Palaysi E Daures JP Pujol JL Cachexia - sarcopenia as a determinant of disease control rate and survival in non-small lung cancer patients receiving immune-checkpoint inhibitors. Lung Cancer (2020) 143:19–26. 10.1016/j.lungcan.2020.03.003 32200137

[113] Takada K Yoneshima Y Tanaka K Okamoto I Shimokawa M Wakasu S Clinical impact of skeletal muscle area in patients with non-small cell lung cancer treated with anti-PD-1 inhibitors. J Cancer Res Clin Oncol (2020) 146(5):1217–25. 10.1007/s00432-020-03146-5 PMC11804506 32025867

[114] Zhou X Yao Z Yang H Liang N Zhang X Zhang F Are immune-related adverse events associated with the efficacy of immune checkpoint inhibitors in patients with cancer? A systematic review and meta-analysis. BMC Med (2020) 18(1):87. 10.1186/s12916-020-01549-2 32306958 PMC7169020

[115] Cortellini A Bersanelli M Santini D Buti S Tiseo M Cannita K Another side of the association between body mass index (BMI) and clinical outcomes of cancer patients receiving programmed cell death protein-1 (PD-1)/ Programmed cell death-ligand 1 (PD-L1) checkpoint inhibitors: A multicentre analysis of immune-related adverse events. Eur J Cancer (2020) 128:17–26. 10.1016/j.ejca.2019.12.031 32109847

[116] Berger A Colpitts SJ Seabrook MSS Furlonger CL Bendix MB Moreau JM Interleukin-15 in cancer immunotherapy: IL-15 receptor complex versus soluble IL-15 in a cancer cell-delivered murine leukemia model. J Immunother Cancer (2019) 7(1):355. 10.1186/s40425-019-0777-8 31856922 PMC6924073

[117] Peddle-McIntyre CJ Singh F Thomas R Newton RU Galvão DA Cavalheri V Exercise training for advanced lung cancer. Cochrane Database Syst Rev (2019) 2(2):CD012685. 10.1002/14651858.CD012685.pub2 30741408 PMC6371641

[118] Zhang H Garcia JM Anamorelin hydrochloride for the treatment of cancer-anorexia-cachexia in NSCLC. Expert Opin Pharmacother (2015) 16(8):1245–53. 10.1517/14656566.2015.1041500 PMC4677053 25945893

